# Risk Factors for Acute Unintentional Poisoning among Children Aged 1–5 Years in the Rural Community of Sri Lanka

**DOI:** 10.1155/2017/4375987

**Published:** 2017-08-08

**Authors:** M. B. Kavinda Chandimal Dayasiri, Shaluka F. Jayamanne, Chamilka Y. Jayasinghe

**Affiliations:** ^1^University Paediatrics Unit, Lady Ridgeway Hospital for Children, Colombo, Sri Lanka; ^2^Faculty of Medicine, University of Kelaniya, Kelaniya, Sri Lanka

## Abstract

**Background:**

Acute poisoning in children is a major preventable cause of morbidity and mortality in both developed and developing countries. However, there is a wide variation in patterns of poisoning and related risk factors across different geographic regions globally. This hospital based case-control study identifies the risk factors of acute unintentional poisoning among children aged 1−5 years of the rural community in a developing Asian country.

**Methods:**

This hospital based case-control study included 600 children. Each group comprised three hundred children and all children were recruited at Anuradhapura Teaching Hospital, Sri Lanka, over two years (from February 2012 to January 2014). The two groups were compared to identify the effect of 23 proposed risk factors for unintentional poisoning using multivariate analysis in a binary logistic regression model.

**Results:**

Multivariate analysis identified eight risk factors which were significantly associated with unintentional poisoning. The strongest risk factors were inadequate supervision (95% CI: 15.4–52.6), employed mother (95% CI: 2.9–17.5), parental concern of lack of family support (95% CI: 3.65–83.3), and unsafe storage of household poisons (95% CI: 1.5–4.9).

**Conclusions:**

Since inadequate supervision, unsafe storage, and unsafe environment are the strongest risk factors for childhood unintentional poisoning, the effect of community education to enhance vigilance, safe storage, and assurance of safe environment should be evaluated.

## 1. Background

Acute poisoning in the pediatric age group is an important cause of preventable mortality and morbidity. The circumstances of poisoning and related risk factors vary widely across different geographic regions globally due to variable accessibility and availability and varied environmental factors. Mortality due to acute unintentional poisoning among children under 4 years of age varies from 0.3 to 7 per 100,000 people in different countries of the world [1]. Accidental poisoning in the pediatric age group is rising day by day in developing countries. Easy availability of household chemicals, medicines, and pesticides predisposes to accidental poisoning. These unintentional self-poisonings have become major health hazards causing severe toxic effects in children with long-term morbidity.

Acute poisoning is an important clinical problem in Sri Lanka and it has a significant economic impact on the health service of the country. The data on financial costs of managing children with unintentional poisoning in rural Sri Lanka, however, are currently unavailable. The significance of this problem lies mainly in the factors predisposing to acute unintentional poisoning. These factors are diverse and include both situational factors (geographic location, social and economic barriers, and culture) and person related factors (personality, lifestyle, parenting style, and education level of parents).

An Asian study [2] showed maternal employment and previous history of poisoning as significant risk factors for unintentional poisoning among children, with unavailability of poisons being a protective factor. The same study reported that poor maternal education, inadequate supervision of children, substance abuse, and mental illness in family members are risk factors that increase the incidence of poisoning in children. The study identified safe storage and health education on prevention of substance abuse as effective interventions for reducing the unintentional poisoning risk among children in the studied community.

The literature on risk factors for acute poisoning among children is sparse in the South Asian region. Ahmed et al. (2011) [3] comprehensively evaluated multiple risk factors of poisoning among children in Pakistan with the focus of their study being on population attributable risk factors for acute poisoning. Agarwal et al. (2016) proposed the need for raising awareness among the public in poisoning prevention based on their epidemiological study in India [4]. Childhood poisoning statistics in Sri Lanka are limited. Though there are a number of studies available for characterizing poisoning patterns among adult populations in Sri Lanka, comprehensive risk factor studies for the pediatric age group are unavailable. A 15-year prospective study (1985–2000) of poisoning patterns among children at Lady Ridgeway Hospital for Children described 2100 children with unintentional poisoning [5]. The study was based predominantly on an urban population. Poisoning trends have changed over the years in the pediatric age group with varied socioeconomic, political, and cultural developments and there have been no studies published in Sri Lanka for more than a decade. The risk factors operating on poisoning patterns are also likely to have changed along with changing trends in childhood poisoning. Availability of such information would indefinitely benefit poison management centers in Sri Lanka in planning preventive interventions, educating the community, and allocating scarce resources more efficiently. In this background, the current study aimed to identify child, caregiver, environment, and poisoning substance related risk factors for unintentional poisoning via a comprehensive analysis of multiple risk factors.

## 2. Methods

### 2.1. Study Setting

This hospital based case-control study was conducted over a period of two years (from February 2012 to January 2014) at Anuradhapura Teaching Hospital. Anuradhapura Teaching Hospital is the largest hospital in the north-central province in which the majority of people belong to a rural community.

### 2.2. Participants

This study involved all inpatient children who presented with acute unintentional poisoning and who were between 1 and 5 years of age. Children were recruited as “cases” after their poisoning events were confirmed by caregivers following the initial evaluation at the hospital's emergency department and subsequently at general pediatric wards. Children with doubtful poisoning and with no clear etiology were excluded from the study. Children who had intentional poisoning were also excluded. Children with past history of poisoning were excluded from both groups. Children with food poisoning, snake envenomation, allergic reactions, and adverse drug reactions which can be considered in the purview of toxicology were also excluded from the study.

Three hundred children were recruited as “cases” over the two-year study period. The “control” group was selected from the same pediatric wards over the same study period. Children, who presented with acute medical illnesses and without any history of chronic medical illnesses or accidental or deliberate poisoning, were interviewed as controls to compare the prevalence of risk factors among the two groups. The acute medical illnesses considered included viral fever, acute upper respiratory tract infection, and urticaria. All other acute conditions including nonspecific symptoms without a definitive diagnosis were excluded. All “cases” were matched for age and gender on individual patient basis. Both groups comprised three hundred children adding to a total of six hundred children. Minimum sample size required for this matched case-control study was 248 pairs (*α*: 0.05, *β*: 0.1, *P*_A_: 0.8, and *P*_D_: 0.1).

### 2.3. Data Collection

Data were collected from the caregivers of children recruited to the “case” and “control” groups. Mothers were interviewed in most encounters and fathers or other caregivers (grandparents/other related caregivers) were interviewed only when mothers were not available to participate in the study. Data collection from all six hundred caregivers was done by the principal investigator himself to minimize interviewer bias. Interviews with the caregivers were conducted on the same day of admission to minimize possible recall bias. Data were collected using a pretested structured questionnaire which comprised questions to identify demographic data, type and circumstances of poisoning, and risk factors for acute poisoning (Appendix) and qualitative evaluation via focused group discussions. The study instruments were pretested by administration of the questionnaire to fifty caregivers (twenty-five in each group) in the same study setting over a two-month period prior to commencement of the study. Risk factors were categorized under four domains, environmental, psychosocial, and family related factors and personal characteristics, and twenty-three risk factors were proposed. Extensive literature survey was done to identify previously reported risk factors in other geographic regions. The investigators proposed and designed a risk factor questionnaire themselves and the questionnaire was administered following careful pretesting and expert review.

### 2.4. Outcome and Exposures

The outcome of interest was medically attended acute poisoning from medicines, household chemical agents, garden plants, and pesticides present at the child's home or home garden resulting in hospital admission. Suspicious and doubtful poisoning events were excluded. Twenty-three proposed risk factors were considered in terms of “exposures.” The proposed 23 risk factors were broadly categorized to environmental, psychosocial, and family related factors and personal characteristics.  Table 1 illustrates the proposed risk factors.

Each risk factor was defined prior to inclusion of those risk factors in the questionnaire.

The presence of childhood personality abnormalities was identified by parents' subjective judgment of the child's personality as being one or more of the following: shy, timid, aggressive, avoidant, antisocial, overdependent, or any psychiatric illness related personality disorder. Child behavioral abnormalities were defined for the study as one of abnormal behaviors including nightmares, night terrors, nail biting, stammering, abnormal eating or sleeping habits, hyperactivity, impulsivity, and attention seeking behavior. The presence of unsafe storage (medicines, household chemicals, and pesticides) was identified when those compounds were not stored in a lockable container or an inaccessible location to the child. Caregivers' judgment was considered in determining the presence of economic and marital problems, sibling related problems, inadequate house space, and lack of family and social support. Harmful alcohol use was considered as an adverse exposure. A young mother was defined as a person who was nineteen years old or less at the time of assessment. The presence of a psychological illness in a parent was defined for the study as being diagnosed with a psychiatric illness or having sufficient clinical criteria for a diagnosis of a psychiatric illness based on DSM-V (Diagnostic and Statistical Manual of Mental Disorders). Inadequate supervision was defined for the study as the lack of consistent presence of a principal caregiver (either mother or father) during the child's stay at the home premises. It was considered as an environmental risk factor based on cultural circumstances given that the home environment and family structure were determining the status of supervision. Incorrect parenting style was defined for the study as one of the nonauthoritative parenting styles including neglectful, permissive, and authoritarian parenting styles.

Developmental delay was defined as a delay of more than six months in achieving milestones in one of four domains of child development up to five years as identified in the Child Health Development Record (CHDR) published by the World Health Organization (WHO). The four domains included gross motor, fine motor and vision, speech, language and hearing, and social and behavioral development. Investigators understood that CHDR provides only crude assessments; however, it was more convenient to use and familiar to the parents of children recruited in the study. Sophisticated developmental assessment tools were not feasible with the study due to limitation of resources and time factor and lack of cooperation from participants.

In order to perform an in-depth analysis of the proposed predisposing risk factors of acute accidental poisoning and to ensure that all the proposed risk factors meet the study definitions, a qualitative study was conducted by the principal investigator himself, recruiting parents of all participant children concurrent with the administration of the pretested, multistructured questionnaire. Each risk factor was evaluated in qualitative terms and in relation to study definitions before the response was recorded in the data collection questionnaire. Data in the qualitative study was recorded as field notes and had emphasis on study definitions. Data collection was done prospectively over two years via focused group discussions.

### 2.5. Statistical Methods and Analysis

All data were analyzed using SPSS version 19.0. All twenty-three proposed risk factors were used to create a binary logistic regression model adjusted for age and gender. In this model, each factor was initially evaluated using univariate analysis for significance levels. Controls were kept as the dependent variables and all proposed risk factors were submitted as categorical covariates. Stepwise backward conditional method was applied in the model. Probability for stepwise method was set as entry 0.5 and removal 0.20. Odds ratios were calculated for each risk factor along with 95% confidence intervals (CI). Independent risk factors were identified in the same model by multivariate analysis. Odds ratios were calculated for each of the risk factors along with 95% confidence intervals for each ratio similar to univariate analysis.

## 3. Results

Six hundred participants comprising 300 children in each of the “case” and “control” groups were available for analysis. All poisoning events occurred by ingestion of a poison. Household poisoning substances were the commonest type of poison (*n* = 102, 34%), followed by medicines (*n* = 88, 29.3%), plant poisons (*n* = 50, 16.7%), pesticides (*n* = 22, 7.3%), and miscellaneous poisons (*n* = 38, 12.7%). The commonest poison was kerosene oil (*n* = 70, 23.3%). Paracetamol (*n* = 22, 7.3%),* Ricinus communis* (*n* = 21, 7%), organophosphate pesticides (*n* = 11, 3.7%), and* Abrus precatorius* (*n* = 10, 3.3%) were other commoner poisons. 234 (78%) poisoning events occurred in either home or home garden. 144 children (48%) were transferred from a regional hospital to Anuradhapura Teaching Hospital.

All children were between 1 and 5 years of age and had similar sex distributions. The age and sex distributions of the two groups are illustrated in Tables 2 and 3.

### 3.1. Univariate Analysis of Individual Risk Factors

Nine out of twenty-three risk factors showed a significant effect at *p* < 0.001 (CI: 99%) in univariate analysis. Those factors included unsafe storage of medicines, unsafe storage of household chemicals, inadequate supervision of the child, mother employment during the daytime, nonauthoritative parenting styles, primary level education in the mother, psychological illnesses in parents, poisonous plants in the home garden, and parental concern of lack of family support. Four risk factors showed a significant effect at 0.001 < *p* < 0.05 (CI: 95%). They included inadequate house space, unsafe use/storage of agrochemicals, developmental delay in the child, and young maternal age (<19 years old). Eight risk factors including history of personality and behavioral disorders in the child, marital problems among parents, mother employment outside the country, parental concern regarding sibling disharmony, lack of schooling/educational opportunities for the child, single parent status, and lack of social support showed no significant association with confidence intervals of respective odds ratios lying on both sides of 1.00. The risk factors observed in univariate analysis are illustrated in Table 4.

### 3.2. Multivariate Analysis in the Binary Regression Model

Table 5 illustrates the independent risk factors observed in multivariate analysis.

In step 8 of the backward conditional approach, eight risk factors were left in the model showing significance of at least *p* < 0.05. The risk factors which showed significance of *p* < 0.05 but >0.001 (confidence level = 95%) included unsafe storage of household chemicals (0.001), nonauthoritative parenting style (0.005), developmental delay in the child (0.012), primary level education in the mother (0.039), and poisonous plants in the home garden (0.001). Three proposed risk factors showed significance of *p* < 0.001 (CI: 99%) and they were inadequate supervision of the child, mother being employed during daytime, and parental concern of lack of family support to look after the child. The lower and upper limits indicating 95% confidence intervals in respective odds ratios in all nine risk factors lied above 1.00.

## 4. Discussion

The current study evaluated a broad range of potential risk factors for unintentional poisoning in children aged 1–5 years. The risk factors were broadly categorized under environmental, psychosocial, and family related factors and personal characteristics.

Unsafe storage of medicines and household chemicals [6], low parental education, low socioeconomic status, larger family size (≥4 children), and history of previous poisoning are previously reported risk factors for acute unintentional childhood poisoning [7, 8]. A recent study concluded that poor child-caregiver relationship is an important risk factor for unintentional poisoning [9]. The current study identified eight independent risk factors for acute unintentional poisoning by multivariate analysis in the binary logistic regression model. The identified risk factors were unsafe storage of medicines, unsafe storage of household chemicals, inadequate supervision of the child, mother employment during the daytime, nonauthoritative parenting styles, primary level education in the mother, poisonous plants in the home garden, and parental concern of lack of family support. Among these factors, the strongest risk factors were inadequate supervision of the child, mother being employed during daytime, and parental concern of lack of family support. Five additional risk factors showed a significant effect in univariate analysis. They were inadequate house space, unsafe use/storage of agrochemicals, psychological illnesses in parents, developmental delay in the child, and young maternal age (<19 years old). Maternal psychiatric illness has been associated with a significantly elevated risk of unintentional poisoning in children in studies from developed countries [9]. Young maternal age [10], unsafe use of pesticides [11], and overcrowding [11] have also been shown to be associated with an increased risk of childhood unintentional injuries. Thus, the current study reinforces previously reported risk factors for unintentional child injuries in different geographic locations.

A European study [8] concluded that the absence of at least one parent was associated with an increased risk of unintentional poisoning. Easy accessibility to the poison increased the risk of toxic exposure in children. It was similarly observed in the current study with inadequate supervision being observed as the strongest risk factor. Unavailability of the mother during daytime and lack of family support in looking after the child were also two of the strongest risk factors recognized in the current study. Brayden et al. pointed out that unintentional poisoning occurs secondary to several factors including unsafe storage of poisons and curiosity of children [12]. The current study in its univariate analysis observed that unsafe storage of medicines, household chemicals, and pesticides was associated with a significantly elevated risk of unintentional ingestion of the respective poisons. The investigators however did not evaluate the child's curiosity due to lack of clear indicators to quantify that factor.

The current study observed no direct or significant association between the economic problems and subsequent poisoning in compliance with other studies [13]. The fact that no significant difference was found between groups with respect to socioeconomic status of families suggests that the magnitude of this variable does not have statistical power. There are other factors that can be attributed to lower socioeconomic conditions such as lack of social and family support, poor maternal education, disruptive family environment, poor child-caregiver relationship, and unsafe storage of poisonous substances which are likely to coexist with poor socioeconomic status.

Unsafe storage of household chemicals and medicines was three and two times more reported among cases than among controls and it was consistent with findings of studies from Pakistan [3], Brazil [14], USA [15], Malaysia, and Thailand [16]. One study [17] from the African continent has noted poor parental education as a risk factor for poisoning. Similarly, the current study appreciated poor maternal education (primary education or below) as one of the strongest risk factors in univariate analysis.

In the current study, inadequate supervision was observed six times more commonly among children with unintentional poisoning compared to the control group, and this is consistent with other Asian studies which reported a fivefold higher risk for unintentional poisoning [18]. European studies also concluded that adequate supervision was the most important factor which prevented childhood poisoning accidents [18], repeatedly highlighting the need for adequate supervision of children who are under five years of age as a measure of preventing unintentional poisoning accidents.

The study did not identify a significant association between childhood behavioral characteristics and acute unintentional childhood poisoning. This was consistent with other studies published from Europe [19]. We observed that less family support was significantly associated with high risks of acute poisoning. This is consistent with findings from other studies in South Asia that concluded that extended family support is protective against unintentional poisoning [3]. Studies show that children, who are poisoned, are more likely to belong to families with few social resources [20, 21]. However, we did not observe lack of social support as a significant risk factor leading to unintentional poisoning.

Other significant findings of the current risk factor study are the implications of the presence of poisoning plants in the home garden, nonauthoritative parenting styles, and developmental delay in the child as major and independent risk factors for acute unintentional poisoning. Evidence from properly controlled studies is scant in the currently available literature regarding these variables and needs further studies. As the majority of unintentional poisoning occurred within home premises in the current study, a holistic approach which targets the household environment would help in managing the burden of poisoning as suggested by other studies [22]. Systematic reviews have proved that provision of safety equipment, home safety education, and heightened community awareness increase safe storage of medicines and household chemicals [23]. Since unsafe storage is among the most significant risk factors, evaluation of the effectiveness of such interventions in this population is worthwhile. The results also show that it is important to build up necessary intersector collaboration to prevent modifiable risk factors through cost-effective interventions, community education, mobilization, and awareness to ensure a safer environment for the child.

This study has several limitations in its methodology. The study was hospital based rather than community based. It is likely that the study has not addressed the poisoning events which were not brought to medical attention during the period of the study. Further, the study was conducted only at Anuradhapura Teaching Hospital which has a wider drainage area within the north-central province of Sri Lanka. Though most children with acute poisoning are transferred from local hospitals to the teaching hospital for further management, a fraction of acute poisoning cases are likely to have been not transferred, thus being not taken into account in the current study. The investigators studied twenty-three risk factors in the case-control study design. The questionnaire underwent expert review, piloting, and evaluation by a psychometrician. However, principal component analysis was not performed and internal consistency was not calculated. Thus, the study instrument may have deficiencies in its validity. The questions were carefully selected following expert review and each risk factor was defined for the study. Data collection from all six hundred participants in the current study was carried out by one investigator and interviews were administered as soon as patients were admitted to the pediatric wards. The authors believe that this likely minimized interviewer bias and recall bias in the questionnaire administration. The sample size was determined for the study based on regional data and most risk factors showed acceptable distributions of 95% confidence intervals of odds ratios; however, given the wide distribution of respective parameters in the variable “inadequate supervision of the child,” the authors suggest reanalysis of the same parameter, recruiting a larger sample to increase reliability.

## 5. Conclusions

Children become victims of acute unintentional poisoning mostly secondary to inadequate supervision by caregivers, unsafe storage of potentially poisonous substances, and unsafe environment. As these risk factors are significantly associated with unintentional poisoning, the effect of community education to enhance vigilance, safe storage, and assurance of safe environment should be evaluated.

## Figures and Tables

**Table 1:** Exposures of interest: the twenty-three proposed risk factors for acute poisoning among children aged 1–5 years.

Risk factor category	Proposed risk factor
(1) Environmental risk factors	(1) Unsafe storage of medicines
	(2) Unsafe storage of household chemicals
	(3) Unsafe use/storage of agrochemicals
	(4) Inadequate space in the house
	(5) Inadequate supervision of the child
	(6) Poisonous plants in the
	neighborhood/home garden

(2) Psychosocial risk factors	(1) Psychological problems in parents
	(2) Lack of social support
	(3) Lack of schooling/education to the child

(3) Family related factors	(1) Father using alcohol or illicit drugs
	(2) Problems with the siblings
	(3) Incorrect parenting styles
	(4) Mother employed outside the country
	(5) Economic problems in the family
	(6) Mother working during the daytime
	(7) Poor education in the mother (<grade 8)
	(8) Young mother (<21 years old)
	(9) Marital problems among parents
	(10) Lack of family support
	(11) Single parent status

(4) Personal characteristics	(1) Developmental problems in the child
	(2) Personality abnormalities in the child
	(3) Behavioral abnormalities in the child

**Table 2:** Age distributions of two groups: “children with acute unintentional poisoning” and “control group.”

Age	Children with unintentional poisoning	Control group
1-2 years old	107 (35.7%)	107 (35.7%)
2–4 years old	153 (51.0%)	153 (51.0%)
4-5 years old	40 (13.3%)	40 (13.3%)

Total	300 (100%)	300 (100%)

**Table 3:** Sex distribution of the two groups with “acute poisoning” and “controls.”

Sex	Children with unintentional poisoning	Control group
Male	169 (56.3%)	169 (56.3%)
Female	131 (43.7%)	131 (43.7%)

Total	300 (100%)	300 (100%)

**Table 4:** Univariate unadjusted analysis of risk factors in the binary logistic regression model.

Proposed risk factor	Cases	Controls	Odds ratio	95% CI (OR)		*p* value
				Low	High	
Environmental risk factors						
(1) Unsafe storage of medicines	142 (47.3%)	72 (24%)	2.85	2.04	4.00	<0.001
(2) Unsafe storage of household chemicals	168 (56%)	54 (18%)	5.80	4.00	8.40	<0.001
(3) Unsafe use/storage of agrochemicals	58 (19.3%)	38 (12.7%)	1.65	1.06	2.57	0.027
(4) Inadequate space in the house	102 (34%)	67 (22.4%)	1.79	1.24	2.57	0.002
(5) Inadequate supervision of the child	249 (83%)	39 (13%)	32.26	20.83	52.60	<0.001
(6) Poisonous plants in the home garden	95 (31.7%)	23 (7.7%)	5.58	3.42	9.09	<0.001
Psychosocial risk factors						
(1) Psychological illness in parents	11 (3.7%)	0	—	—	—	<0.001
(2) Lack of social support	43 (14.3%)	42 (14%)	1.02	0.64	1.62	0.907
(3) Lack of schooling/education to the child	5 (1.7%)	7 (2.3%)	0.70	0.22	2.26	0.562
Family related risk factors						
(1) Father using alcohol or illicit drugs	61 (20.3%)	87 (29%)	0.62	0.43	0.91	0.014
(2) Sibling disharmony	12 (4%)	18 (6%)	0.65	0.31	1.38	0.260
(3) Nonauthoritative parenting styles	35 (11.7%)	2 (0.7%)	19.6	4.69	83.3	<0.001
(4) Mother employed outside the country	2 (0.7%)	5 (1.7%)	0.40	0.08	2.06	0.270
(5) Economic problems in the family	115 (38.3%)	155 (51.7%)	0.58	0.42	0.81	0.001
(6) Mother working during the daytime	68 (22.7%)	14 (4.7%)	5.98	3.28	10.87	<0.001
(7) Primary level education in the mother	51 (17%)	15 (5%)	3.89	2.13	7.09	<0.001
(8) Young mother (<19 years old)	37 (12.3%)	19 (6.3%)	2.08	1.16	3.70	0.013
(9) Marital problems among parents	23 (7.7%)	13 (4.3%)	1.83	0.91	3.69	0.090
(10) Lack of family support	122 (40.7%)	42 (14%)	4.20	2.82	6.28	<0.001
(11) Single parent status	2 (0.7%)	3 (1%)	0.67	0.11	4.00	0.656
Personal characteristics						
(1) Developmental delay in the child	18 (6%)	3 (1%)	6.31	1.84	21.68	0.003
(2) Personality abnormalities in the child	3 (1%)	2 (0.7%)	1.51	0.24	9.09	0.656
(3) Behavioral abnormalities in the child	6 (2%)	2 (0.7%)	3.03	0.61	15.10	0.175

**Table 5:** Multivariate analysis of independent risk factors for acute unintentional pediatric poisoning in the binary logistic regression model.

Proposed risk factor	Odds ratio	95% CI (OR)		*p* value
		Low	High	
Environmental risk factors				
(1) Unsafe storage of household chemicals	2.70	1.49	4.90	0.001
(2) Inadequate supervision of the child	28.50	15.38	52.60	<0.001
(3) Poisonous plants in the home garden	3.67	1.70	7.93	0.001
Family related risk factors				
(1) Mother working during the daytime	7.14	2.89	17.54	<0.001
(2) Nonauthoritative parenting styles	12.34	2.14	71.40	0.005
(3) Primary level education in the mother	2.73	1.05	7.14	0.039
(4) Lack of family support	17.54	3.65	83.3	<0.001
Personal characteristics				
(1) Developmental delay in the child	7.93	1.57	40.00	0.012

## Appendix

### Quantitative Data Collection Study Instrument

*Risk Factors for Acute Unintentional Poisoning among Children in Rural Community of Sri Lanka*

  Date of data collection: …/…/…
  Hospital: …
  Date of admission: …/…/…
  Ward: …

*Part 1*

* (1) Basic Demographic Data*

1. Name: …
2. BHT Number: …
3. Age: …
4. Gender: male/female
5. Residential address: …
6. Medical officer of health (MOH) division: …
7. Public health midwifery (PHM) division: …
8. Parent's education level: Father: … Mother: …
9. Parents' occupation: Father: … Mother: …
10. Ethnicity: … Religion: …

*Part 2*

* (2.1) Reason for Hospital Admission*

…

*(2.2) Type of Poison*. Household poisons/medicines/poisonous plants/agrochemicals/miscellaneous

(2.2.1) General/trade name of the poison: …

(2.2.2) Chemical/scientific name of the poison (if available): …

*Part 3*

(3.1) Quantity of poison: …
(3.2) Location of the poisoning event: …
(3.3) Route of poisoning: ingestion/inhalation/direct skin contact/other
(3.4) Transferred hospital:…/not applicable

*Part 4*

(4.0) Proposed risk factors
    Present
    Absent
(1) Inadequate space in the house
    Present
    Absent
(2) Nonauthoritative parenting styles
    Present
    Absent
(3) Unsafe use/storage of agrochemicals
    Present
    Absent
(4) Lack of schooling/education to the child
    Present
    Absent
(5) Inadequate supervision of the child
    Present
    Absent
(6) Unsafe storage of household chemicals
    Present
    Absent
(7) Young mother (<19 years old)
    Present
    Absent
(8) Marital problems among parents
    Present
    Absent
(9) Sibling disharmony
    Present
    Absent
(10) Mother employed outside the country
    Present
    Absent
(11) Father using alcohol or illicit drugs
    Present
    Absent
(12) Unsafe storage of medicines
    Present
    Absent
(13) Mother working during the daytime
    Present
    Absent
(14) Economic problems in the family
    Present
    Absent
(15) Single parent status
    Present
    Absent
(16) Personality abnormalities in the child
    Present
    Absent
(17) Behavioral abnormalities in the child
    Present
    Absent
(18) Primary level education in the mother
    Present
    Absent
(19) Lack of family support
    Present
    Absent
(20) Psychological illness in parents
    Present
    Absent
(21) Lack of social support
    Present
    Absent
(22) Developmental delay in the child
    Present
    Absent
(23) Poisonous plants in the home garden
    Present
    Absent
